# Disposable DNA Amplification Chips with Integrated Low-Cost Heaters †

**DOI:** 10.3390/mi11030238

**Published:** 2020-02-25

**Authors:** Henk-Willem Veltkamp, Fernanda Akegawa Monteiro, Remco Sanders, Remco Wiegerink, Joost Lötters

**Affiliations:** 1 Department of Integrated Devices and Systems, University of Twente, P.O. Box 217, 7500 AE Enschede, The Netherlands; nanoakegawa@gmail.com (F.A.M.); r.g.p.sanders@utwente.nl (R.S.); r.j.wiegerink@utwente.nl (R.W.); j.c.lotters@utwente.nl (J.L.); 2 Bronkhorst High-Tech BV, Nijverheidsstraat 1A, 7261 AK Ruurlo, The Netherlands

**Keywords:** early-stage disease detection, multiple displacement amplification, on-chip DNA amplification, integrated heaters, cyclic olefin copolymer, polymer-based disposable microfluidic chips, zoonoses

## Abstract

Fast point-of-use detection of, for example, early-stage zoonoses, e.g., Q-fever, bovine tuberculosis, or the Covid-19 coronavirus, is beneficial for both humans and animal husbandry as it can save lives and livestock. The latter prevents farmers from going bankrupt after a zoonoses outbreak. This paper describes the development of a fabrication process and the proof-of-principle of a disposable DNA amplification chip with an integrated heater. Based on the analysis of the milling process, metal adhesion studies, and COMSOL MultiPhysics heat transfer simulations, the first batch of chips has been fabricated and successful multiple displacement amplification reactions are performed inside these chips. This research is the first step towards the development of an early-stage zoonoses detection device. Tests with real zoonoses and DNA specific amplification reactions still need to be done.

## 1. Introduction

Diseases were and can still be a major problem in the world. Examples are outbreaks of zoonoses. One very recent example is the Covid-19 coronavirus outbreak in the People’s Republic of China. Zoonoses are also a widespread problem in animal husbandry [1]. This group encompasses diseases which can be transferred between animals (usually vertebrates) and between animals and humans. They are transmitted through zoonotic agents (e.g., bacteria, viruses, fungi, and parasites) [2,3,4]. Examples of bacterial zoonoses are the infections caused by *Coxiella burnetii* (Q-fever), *Mycobacterium bovis* (bovine tuberculosis), and by species of the Salmonella (Salmonellosis), Campylobacter (Campylobacteriosis), and Escherichia (Escherichiasis) genus [2,5]. These diseases are of potential risk for humans and livestock of farms. Poon et al. show that early-stage detection of coronaviruses positively influence the survival chances of patients [6]. An outbreak among the livestock of a farm is often disastrous to the owner of that farm, and for people living in the proximity of that farm [7]. Often, more animals of the livestock are infected and the whole livestock is exterminated out of precaution, which could lead to bankruptcy of the farmer. Therefore, early-stage detection of this group of diseases, and other diseases as well, is often the key to save lives and livestock. As these diseases are also encountered at remote locations and in developing countries, it is desired that such detection equipment is portable and as cheap as possible. A lab-on-a-chip platform can be used for this early-stage detection.

In the early stage of diseases, the agent, and therefore its genetic material, is only present in low concentrations within the infected human or animal, making detection rather difficult. One way to overcome this low concentration is to amplify the genetic material of the agent, i.e., deoxyribonucleic acid (DNA) in case of bacteria and DNA or ribonucleic acid (RNA) in case of viruses, until a certain threshold is reached and detection of the disease is made possible. When this amplification reaction is specific to certain DNA or RNA sequences, for example, by using polymerase chain reaction (PCR) [6,8], helicase-dependent amplification (HDA) [9,10], or loop-mediated isothermal amplification (LAMP) [11], and when a fluorescent DNA or RNA binding dye is used, a simple yes-or-no answer for a specific disease can be obtained.

### 1.1. State-of-the-Art

In the past, several chip-based DNA and RNA amplification devices are reported. It goes beyond the scope of this paper to discuss the state-of-the-art of DNA amplification chips in-depth. There are several good review papers written on this topic [12,13,14,15,16,17,18,19,20]. Readers are referred to these for a comprehensive overview of the field. In this paper, the state-of-the-art is divided into several discussion points, i.e., the heating method, the temperature control method, and the substrate material and fabrication technique. These points will be discussed separately.

With respect to heat supply, different methods have been employed. Almassian et al. give a comprehensive overview of different possible heating methods in their review paper [12]. Not all of the mentioned methods are easy to implement in low-cost and portable lab-on-a-chip devices due to their bulkiness or implementation costs. Examples of these rather difficult methods are using heating via induction, infrared, or microwave radiation. Others are not useful due ot their challenging temperature control, like with heating up the system using exothermic reactions. Within the field of DNA amplification, different mechanisms of amplification exist. Some are based on thermo cycling processes, e.g., PCR, whereas others are isothermal. The use of an isothermal amplification technique puts less requirements on the heaters. Isothermal processes are either truly isothermal or consisting of three different temperatures, as they have a thermal denaturation step before and a termination step after the elongation step. The switching between these temperature steps does not have to be as fast as with thermal cycling steps in, for example, PCR amplification reactions. The use of less temperature variations makes it easier to maintain the set temperature as there is less heating an cooling involved. Furthermore, it eliminates the use of a continuous flow approach in systems with low thermal conductivities, e.g., polymers. Therefore, it is easier to implement within lab-on-a-chip devices [17,21]. Isothermal DNA amplification reactions can already be performed by putting the chip on a commercially available hotplate [22,23] or Peltier elements [24,25]. However, these heating systems are bulky and power-consuming. Therefore, they are not useful for portable equipment or operation at remote locations. Miniaturizing heaters lowers the bulkiness and power consumption. Miniaturized heaters can be integrated as integrated resistive heaters, e.g., as deposited thin-film metal [26,27,28,29] or as laminated Cu foil [30], or as micro-Peltier elements [31,32]. These miniaturized heaters can be implemented directly onto the microfluidic chip [28] or on a different substrate and leter incorporated onto the microfluidic chip [33,34,35,36]. The geometry of such a heater contributes significantly to the uniformity of the heat distribution within the chip [26,37]. One method to accurately control the temperature is the use of a proportional-integral-derivative (PID) controlled thermostat. These PID controllers are coupled to the electrical heaters and use a thermocouple as feedback-loop to the controller [22,23,24,25].

There are various materials that can be used to fabricate lab-on-a-chip devices for DNA or RNA amplification. In the past 15 years, more than ten polymers, ceramic materials, and metals have successfully been used to fabricate such devices [15]. The major property playing a role here is the biocompatibility of the material. The surface of the microfluidic structure should not inhibit the amplification reaction. This biocompatibility can be an intrinsic property of the material or the surface can be modified or coated to achieve this [12,13,14,15,16,17,18,19,20]. One often used material is polydimethylsiloxane (PDMS) [22,23,31,32,34,35,38], which can be processed using soft lithography [39]. However, this is a fabrication technology used in academia and is not suitable for upscaling to mass production [40]. Fabrication methods suitable for mass production are thermoforming/embossing or injection molding [41]. One of the materials which is biocompatible and suitable for both industrial scale fabrication technologies is cyclic olefin copolymer (COC) [42], which is one of the materials used in the past as well [28,36,43,44,45]. Guckenberger et al. estimates the costs of injection molding of only 50 simple microfluidic devices on $47, but this becomes cheaper when the mass production stage is reached [41]. Another benefit of COC is the possibility to shape it using micromilling. This technique is a rapid prototyping technology and therefore very useful within proof-of-concept projects [41].

Integrating resistive metal tracks onto a COC substrate have also been done in the past. Some papers describe the use of a surface modification step done before metal deposition in order to enhance adhesion between the COC and the metal layer, like a pretreatment with plasma [46] or an organic solvent [47]. Other papers describe the direct deposition of metal onto the COC surface [28,48]. Chung et al. specifically, fabricated an amplification chip in COC with integrated Au heaters [28]. However, their system required heating from both sides as the used grade of COC has a glass transition temperature ($T_{g}$) of 130 °C. This COC could not withstand the required heater temperatures to have enough heat flux into the system. They had to heat up the heater to temperatures above 130 °C, which caused cracking of the heater tracks due to deformation of the COC. With their double-sided heating they ensured that the reaction mixture had the desired PCR temperatures. However, double-sided heating doubles the amount of metal required, increases the amount of fabrication steps, and therefore increases the price per chip.

### 1.2. The Presented Work

The work presented at the 4th Microfluidic Handling Systems conference and which is extended in this paper aims at the development of a disposable, polymer-based DNA amplification lab-on-chip system with integrated resistive heater based on the World Health Organization (WHO) Sexually Transmitted Diseases Diagnostics Initiative (SDI) ASSURED criteria. Devices which are ASSURED are (A) affordable, (S) sensitive, (S) specific, (U) user-friendly, (R) robust and rapid, (E) equipment-free, and (D) deliverable to those who need them [20,49]. The first step towards such a device is the development of the chip itself. This paper focuses on the choice of substrate material, metal deposition method, and type of metal. Although, it is mentioned above that PCR and HDA are sequence specific, the reaction chosen is the isothermal multiple displacement amplification (MDA) [50]. This reaction is more straightforward [51], as it amplifies any present DNA, and is therefore better suitable as a proof-of-principle amplification reaction to show the functioning of the integrated heater and the biocompatibility of the substrate after the fabrication process. The use of an isothermal amplification technique also simplifies the final device and lowers its footprint, as there are no pumps required. In this research, external analysis methods are used which do not contribute to the WHO-SDI ASSURED criteria due to their bulkiness, costs, and difficulty. However, suggestions and comments on the integration of low-cost detection methods, which are ASSURED, are given in Section 5.

### 1.3. Multiple Displacement Amplification

**Figure 1:**
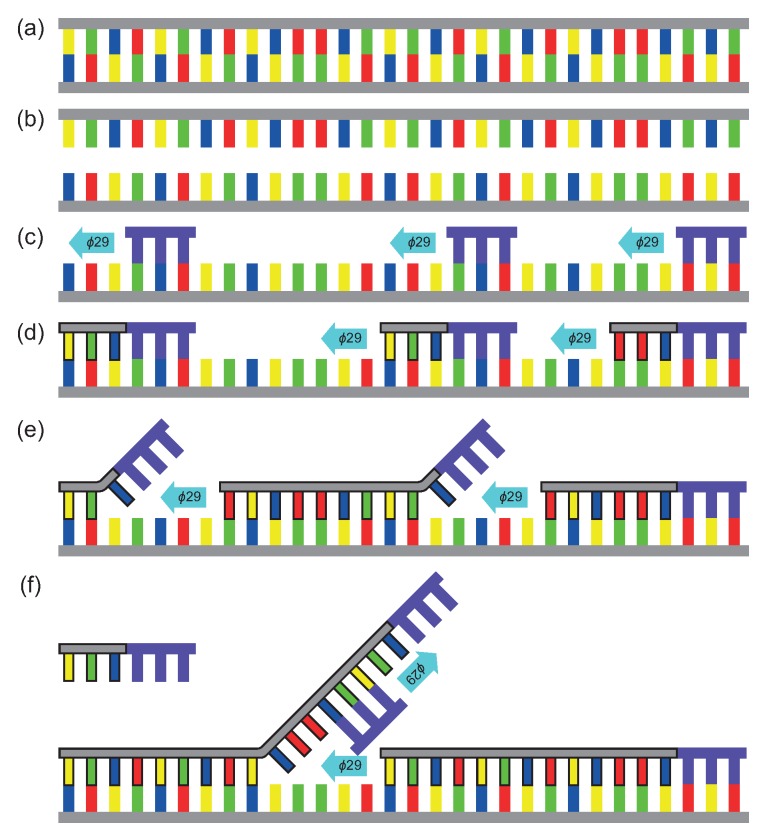
Schematic representation of the MDA reaction. In panel (**a**), a dsDNA strand is shown. In panel (**b**), denaturation happens at 95 °C. In panel (**c**), the random hexamer-primers (purple) and ϕ29 DNA polymerase (blue arrow) bind to the initiation sites. In panel (**d**), the amplification is performed by the polymerase, which binds complementary bases to the ssDNA strand. In panel (**e**), the polymerase encounters another hexamer binded to an initiation site and starts lifting up this hexamer. In panel (**f**), a hexamer binds to the displaced ssDNA strands and the polymerase starts the amplification from this initiation site. For clarity, the amplified DNA is bordered with black.

The proof-of-principle amplification of choice is a MDA reaction, which is a non-specific isothermal method of amplification performed around 30 °C [50]. MDA is a method of whole genome amplification (WGA), as it amplifies all present DNA [52]. It is commonly used when the initial amount of DNA sample is very low. After the WGA is performed, a sequence specific amplification can be done since the quality the amplified DNA by MDA is very high [53]. The amplification reaction is illustrated below in Figure 1 (the contour of the amplified sequence is highlighted in black for clarity). Starting with a double stranded DNA (dsDNA) molecule, a denaturation step at 95 °C is required, giving the random hexamer-primers and the ϕ29 DNA polymerase access to the bases of single stranded DNA (ssDNA) strands. The hexamers anneal themself to aleatory parts of the ssDNA sequence. These hexamers work as initiation sites for the ϕ29 DNA polymerases. After denaturation at 95 °C, the mixture is cooled down to ice temperature and the rest of the reagents are added. The mixture is heated up to ~30 °C so the polymerase starts to complete the complementary ssDNA sequence, creating again a dsDNA strand, eventually it encounters a hexamer from another annealing site. Once this happens the polymerase will lift up that hexamer and starts to separate the amplified sequence formed from that annealing site. As the polymerase displaces the formed strand ahead of it, it continues to complete the sequence. The displaced strand becomes a new ssDNA strand and therefore, it gives new sites for more primers to attach and initiation sites for the polymerase, continuing the amplification, and thus creating a web of DNA strands. Finally, the inactivation of the polymerase is done by heating up the system to 65 °C.

Even though MDA is considered an isothermal process, prior to the reaction and to the addition of most reactants, the dsDNA and a buffer are heated up to 95 °C to denature the dsDNA to ssDNA and to give hexamers the initial access to the ssDNA. After the amplification reaction, the polymerase has to be inactivated at 65 °C. However, this does not require fast temperature changes, as would be the case with, for example, the temperature cycling in PCR amplifications. This, together with the robustness of the amplification (it is a self-limiting reaction that amplifies all present DNA [50]) makes MDA perfectly suitable as proof-of-principle amplification reaction for such devices.

## 2. Design and Fabrication

### 2.1. Microfluidic Structure Design

**Figure 2:**
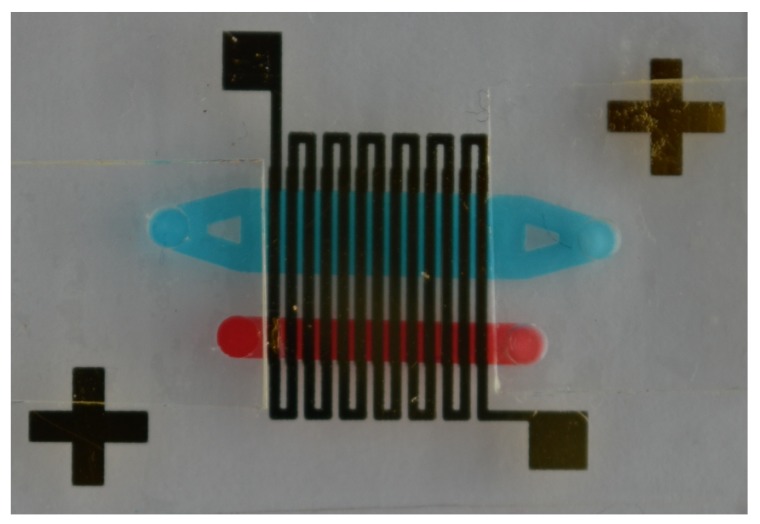
Close-up of the chip showing the Au resistive heater and alignment crosses, the DNA amplification chamber filled with blue food coloring dye, and the temperature monitor chamber filled with red food coloring dye.

**Figure 3:**
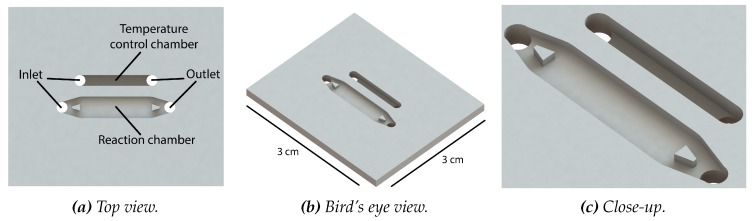
The SolidWorks design of the DNA amplification chip with reaction chamber and chamber for thermocouple-assisted real-time temperature monitoring. This drawing is used to set up the code for the CNC mill. Total size of the chip is 3 cm by 3 cm. Panel (**a**) shows the top view, panel (**b**) shows the bird’s eye view, and panel (**c**) the close-up of the chambers with the in-chamber trapezoid structure.

**Figure A1:**
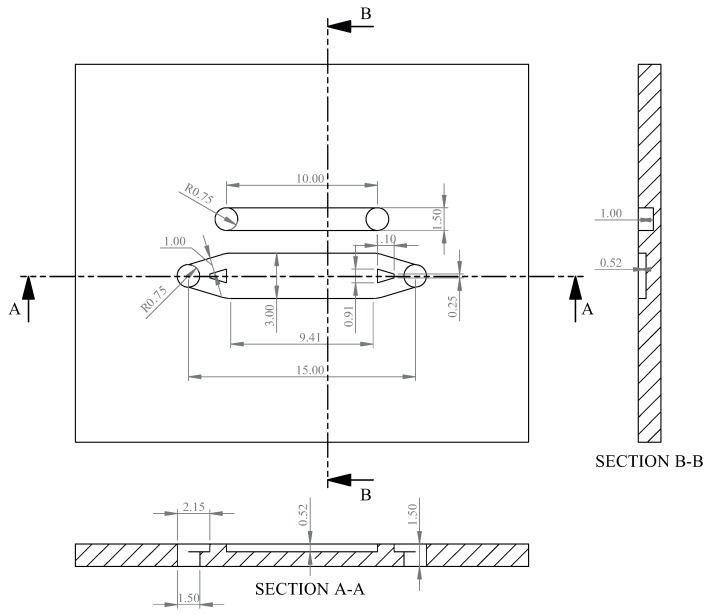
Technical drawing of the DNA amplification chip. All dimensions are in mm. The total chip size is 3 mm by 3 mm.

The microfluidic structure consists of two chambers, i.e., a reaction chamber and a temperature monitor chamber. In Figure 2, a close-up of the final chip is shown. For clarity reasons, the two microfluidic structures are colored with food coloring dye. The reaction chamber is based on the work of Bruijns et al. [36] and its dimensions are chosen in such way that the internal volume of the reaction chamber is the same as the reaction volume of the used Illustra GenomiPhi V2 DNA amplification kit (GE Healthcare Life Sciences, Eindhoven, The Netherlands) together with the EvaGreen dye solution (Biotium, Fremont, CA, USA), while maintaining an as low as possible surface-area-to-volume ratio [44]. Using SolidWorks 2018 computer-aided design (CAD) software (Dassault Systemes, Vélizy-Villacoublay, France), the 3D image of the chip is drawn and with the use of the AutoDesk HSMWorks computer-aided manufacturing (CAM) plug-in (Autodesk Inc., San Rafael, CA, USA), this image is transferred into a computer numerical control (CNC) milling code. The total chip size is 3 cm by 3 cm and contains an inlet and outlet of 1.5 mm diameter. The inlet and outlet are of such size that the reaction chamber can be filled using pipette tips. In between the inlet and outlet, a rectangular reaction chamber of 10 mm by 3 mm is located. Two trapezoid structures are placed in the tapered channels between the inlet/outlet and the chamber. The function of these trapezoids is twofold: First, they minimize the dead volume between the inlet/outlet and the reaction chamber, locating as much as possible of the reaction mixture inside the chamber. Second, they provide support for the chamber closure. A stadium-shaped channel of 1.5 mm wide and 1.0 mm deep is located next to the reaction chamber, in such way that this channel is also covered by the heater. This channel serves as temperature monitor chamber. A thermocouple is inserted in this channel for real-time monitoring of the temperature inside the chip. This way, a more accurate temperature of the reaction mixture inside the chip can be obtained. Via a feedback loop, the input potential can be changed when required. In Figure 3, the SolidWorks design of the chamber-based chip with both chambers is shown. In Figure A1, in Appendix A, the technical drawing of the chip can be found.

### 2.2. Resistive Heater Structure Design

A resistive heater structure will be placed at the bottom side of the chip using shadow masks and a metal deposition method capable of being used for large-scale production. A meandering heater design is chosen, as this minimizes the input power required to heat up the heater. This is evident from Equation (1), which is the relation between Joule’s law, Ohm’s law, and Pouillet’s law.

(1) $$P=\frac{A_{cross-section}*V^{2}}{\rho_{res,i}*l_{heater}}$$

Here, *P* is the input power, $A_{cross-section}$ is the cross-sectional area of the resistor, *V* is the input potential, $\rho_{res,i}$ is the resistivity of resistor material *i*, and $l_{heater}$ is the length of the resistor. This makes a meandering structure, or any other narrow line structure, a quite often used pattern for heaters or electrodes within micro-electromechanical structures and microfluidics [26,29,54,55].

MDA is being done at temperatures of around 30 °C [50], which is lower than, for example, temperatures required for HDA (64 °C) [9] or LAMP (65 °C) [11] and the required PCR temperatures of Chung et al. (95 °C, 54 °C, and 72 °C) [28]. However, most amplification methods require a DNA denaturation step at 95 °C. Equation (2) is used to make an estimation of the required heating powers for a COC–H_2_O–COC stack (in the real device, the upper plate is an adhesive PCR foil, but the thermal properties of this foil are unknown).

(2) $$P=\frac{\Delta T}{R_{th}}=\Delta T*A_{heated}*\left(\frac{\kappa_{COC}}{l_{COC,1}}+\frac{\kappa_{H_{2}O}}{l_{H_{2}O}}+\frac{\kappa_{COC}}{l_{COC,2}}+h\right)$$

Here, $R_{th}$ is defined as the sum of all thermal resistances in series:

(3) $$R_{th}=\frac{1}{h\times A_{heated}}+\sum_{i}^{substances}\left(\frac{l_{i}}{\kappa_{i}\times A_{heated}}\right)$$

Here, *P* is the required power, ΔT is the temperature difference, $R_{th}$ is the thermal resistance, $A_{heated}$ is the heated area, *h* is the convective heat transfer coefficient (being 10 W m^−2^ K^−1^ for convection to air [56]), $\kappa_{i}$ is the thermal conductivity of substance *i*, and $l_{i}$ is the thickness of substance *i*. Values for $\kappa_{i}$ can be found in Appendix B. From Equation (3), the product $R_{th}\times A$ can be defined as the sum of 1/h and $l_{i}\;/\;\kappa_{i}$. Based on this summation, one can conclude that the convective heat transfer to the air is the most present heat transfer mechanism within the system (begin almost a factor 100 higher than the heat lost in the COC and H_2_O). This is also evident from solving Equation (2) for every individual temperature differences within the system and also including convective heat transfer directly from the heater into the air. If a heated area of 7.7 mm by 10.1 mm is assumed, which covers both the reaction chamber and the temperature monitor chamber, and a system consisting of 1 mm COC–0.5 mm H_2_O–0.1 mm COC is assumed, than the heater temperatures and powers in Table 1 are required. These are all in the workable range when a COC of a proper grade is chosen (e.g., TOPAS 6017 has a $T_{g}$ of 170 °C). The only side note here is that at higher temperatures, the temperature gradient through the system also becomes larger. This can be eliminated by using double-sided heating, like Chung et al. [28].

To determine the optimal heater width and heater spacing in the heated area, a parametric study using COMSOL Multiphysics 5.3a finite element method simulations with the *Heat Transfer in Solids* (ht) package is done (COMSOL Inc., Burlington, MA, USA). The model is designed such that it consists of two parallel rectangles of COC (in the real device, the upper plate is an adhesive PCR foil) with H_2_O in between. The meandering heater are assumed to be lines at the bottom side of the layer stack. This reduces the required complexity of the mesh tremendously, as the heater in the real device will be approximately 100 nm in thickness. The heater temperature is set at a constant temperature of 303.15 K. This makes the heater material independent and the model purely focused on the heat transfer inside the COC–H_2_O–COC stack. All used values and equations are given in Appendix B. The layer stack is meshed with an extremely fine mapped mesh consisting of 280.650 elements with average quality of 0.9966. A parametric sweep from 0.3 mm to 2.0 mm, in steps of 0.1 mm, is done for both the heater width ($w_{heater}$) and the heater spacing ($s_{heater}$), giving 324 combinations. The simulations are solved by using the fully coupled, direct Pardiso solver on a custom-build and 40% CPU overclocked simulation computer, containing the equipment listed in Table 2.

To validate whether the metal tracks can withstand the required current, a quick analysis is done for the four extreme cases (i.e., $w_{heater}$ of 0.3 mm and 2.0 mm and $s_{heater}$ of 0.3 mm and 2.0 mm). In the same heated area of 7.7 mm by 10.1 mm a 100 nm ($t_{heater}$) thick heater track consisting of rectangles is assumed. The total amount of large and smaller interconnecting rectangles for all 4 cases is estimated in Table 3. Based on the polynomial approximation equations for the resistivity of Au and Pt ($\rho_{res,i}$, where *i* is either Au or Pt) which COMSOL MultiPhysics 5.3a uses (Equations (4) and (5)) and Equation (6) an estimation is made for the required input currents and the created current densities (defined as $I_{i}\;/\;A_{cross-section}$, in A m^−2^) when the heater is operated at 129.4 mW to get a temperature of 95 °C. These estimations are also given in Table 3.

(4) $$\begin{array}{cc} \rho_{res,Au}\left(T\right)= & -2.210068\times 10^{-9}+9.057611\times 10^{-11}*T\;\;\;\mathrm{for}\;60\leq T<400\\ \; & -4.632985\times 10^{-14}*T^{2}+6.950205\times -17*T^{3} \end{array}$$

(5) $$\begin{array}{cc} \rho_{res,Pt}\left(T\right)= & -1.927892\times 10^{-8}+5.233699\times 10^{-10}*T\;\;\;\mathrm{for}\;160\leq T<600\\ \; & -4.107885\times 10^{-13}*T^{2}+6.694129\times -16*T^{3}\\ \; & -4.447775\times 10^{-19}*T^{4} \end{array}$$

(6) $$I_{i}=\sqrt{\frac{P}{R_{i}}}=\sqrt{\frac{P*A_{cross-section}}{\rho_{res,i}*l_{heater}}}$$

In which $\rho_{res,i}$ is the resistivity, *T* is the temperature, $I_{i}$ is the current going through the resistor, $P_{i}$ is the input power, *R* is the resistance of the resistor, $l_{heater}$ is the length of the resistor, and $A_{cross-section}$ is the cross-sectional area of the resistor defined as width times thickness ($w_{heater}\times t_{heater}$). The subscript *i* denotes the material, being Au or Pt.

All these current densities are below the critical current densities for Au and Pt, which are around 10^10^ A m^−2^ [57] and 10^11^ A m^−2^ [58], respectively. Therefore, any possible combination of heater width and heater spacing will give a resistive that can withstand its operation.

### 2.3. Fabrication

**Figure 4:**
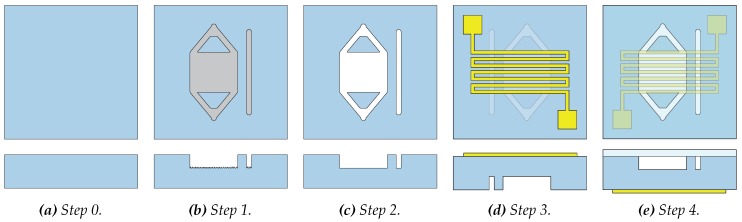
Schematic top views (top row) and cross-sectional representations (bottom row) of the full fabrication process. In panel (**a**) the pristine COC substrate is shown as step 0. Then, in panel (**b**) the first step is shown, i.e., the milling of the reaction chamber and temperature monitor chamber. In panel (**c**), the cyclohexane vapor post-treatment is shown. Then, the substrate is flipped and metal is deposited as step 3 in panel (**d**). The substrate is flipped back and the microfluidic structure is closed using PCR foil in panel (**e**). The images are not on scale and out of proportion for clarity reasons.

COC [42] is chosen as polymeric substrate because of its biocompatibility, optical transparency, physical resistance, chemical resistance, electrical insulation, and price. This copolymer consists of two monomers, an apolar bridged cyclic hydrocarbon (norbornene) monomer and a linear, lesser apolar, linear ethene monomers. Injection molded COC plates (10 cm by 10 cm and 1.5 mm thickness) of the grade TOPAS 6017 (see Figure 4a) are obtained via Kunststoff-Zentrum Leipzig (Kunststoff-Zentrum gGmbH, Leipzig, Germany). This grade is chosen because of its high norbornene content, giving it a relatively high $T_{g}$ of 170 °C. This minimizes the chance of melting during the milling process and decreases the chance of heater failure due to a deforming substrate during operation of the heater [28]. The microfluidic structure explained in Section 2.1 is CNC-milled using a Mikron WF 21C milling machine (Mikron SA Agno, Agno, Switzerland), as can be seen in Figure 4b. Milling is a very fast prototyping technique and chosen because of its flexibility [41]. The milling creates a surface roughness, which increases the surface-area-to-volume ratio. This roughness increases the chance of inhibition during the amplification due to the interaction of the used chemicals with the surface [44]. It also causes a considerable loss of optical transparency, which could obstruct the potential use of in situ fluorescence detection in future devices. Therefore, a chemical post-treatment with cyclohexane vapor is done (see Figure 4c). Such treatment dissolves a thin outer layer of the COC substrate and causes reflowing of the surface roughness due to the surface tension of the material, restoring the optical transparency and reducing the surface roughness [59].

CNC milling and subsequent cyclohexane vapor post-treatment are less suitable for mass production. However, COC has the possibility of being injection molded [42]. The used substrates are made using this method. This is a large-scale production method and could lower the costs of the eventual product and it eliminates the cyclohexane vapor post-treatment, as injection-molded chips would have the same optical transparency as the pristine substrates. Guckenberger et al. mention production costs of $ 47 per simple microfluidic device when only 50 pieces are fabricated [41]. This price is expected to drop drastically when large numbers are fabricated.

A metal is deposited on the backside of the substrate using two laser-cut metal (Mo) shadow masks to outline the shape of the resistive heater (see Figure 4d). Mo has a smaller coefficient of thermal expansion than stainless steel, and therefore gives less deformation during the deposition. Metals of interest are Au or Pt, which are commonly used metals to function as resistive heaters [54]. The deposition methods studied are DC magnetron sputtering using a custom-build machine (Techno Centrum voor Onderwijs en Onderzoek, University of Twente, Enschede, The Netherlands) and e-beam physical vapor deposition (evaporation) using a Balzers BAK 600 CE (Oerlikon Balzers limited, Balzers, Principality of Liechtenstein). Both deposition methods are capable of large-scale production, which will lower the production costs in the large-volume production stage. The metal and deposition method will be chosen based on the metal adhesion performances on the COC substrate, which is studied using the Scotch tape test [60,61], and the resistance versus temperature behavior in the range 20 °C to 100 °C, which is measured in a Heraeus T5025 oven (Heraeus Holding GmbH, Hanau, Germany), customized with electrical readout and connected to a custom-build National Instruments LabVIEW program (Austin, TX, USA).

### 2.4. Operation

The chambers with the resistive heater on the backside, are intensively cleaned by rinsing with acetone, MilliQ DI water, ethanol, and isopropanol [45]. Each cleaning step was done 3 times and the chips are blow dried using N_2_ gas. After drying, the chambers are closed using Microseal “B” PCR plate sealing foil from Bio-Rad (Bio-Rad Inc., Hercules, CA, USA), which is cut in the proper size and manually attached on top of the substrate (see Figure 4e). The DNA, reactants and buffer solutions from the Illustra GenomiPhi V2 DNA amplification kit and an EvaGreen fluorescence dye are pipetted inside the chip using the inlet aperture, after which the inlet and outlet are closed using the same PCR foil. An input potential is applied on the resistive heater using a Keithley 2602 SYSTEM SourceMeter (Cleveland, OH, USA) until they acquire the desired temperature for the amplification. The temperature is real-time monitored by inserting a 162 series RS Technics thermocouple K (RS Components B.V., Haarlem, The Netherlands) in the temperature monitor chamber. The thermocouple is read out with a Tenma 72-7715 Thermometer (Premier Farnell Ltd., Leeds, UK). The source and the read-out of the thermocouple are operated using a custom-programmed LabVIEW program. The initial potential is based on the heater characterization measurements, but will be adjusted according to the feedback-loop of the thermocouple. Detection of the amplification is done *ex-situ* by using quartz cuvets and an Horiba Scientific FluoroMax+ spectrofluorometer (Horiba Scientific, Piscataway, NJ, USA).

## 3. Results and Discussion

### 3.1. COMSOL MultiPhysics Simulation Results

**Figure 5:**
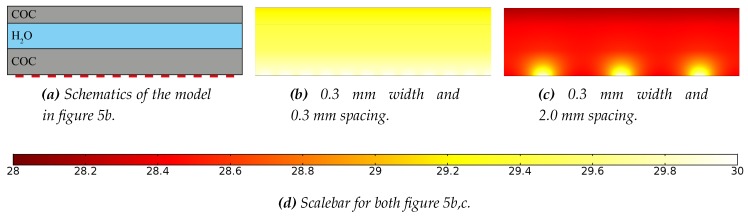
COMSOL Multiphysics 5.3a finite element method heat transfer simulations of a 0.75 mm deep chamber with different heater widths and heater spacings. Panel (**a**) shows the schematics with the materials indicated in the figure and the heaters exaggerated in red (they are 1 dimensional lines in the simulation model). Panel (**b**) shows the case for 0.3 mm heater width and 0.3 mm heater spacing. Panel (**c**) shows the case for 0.3 mm heater width and 2.0 mm heater spacing. The scale bar in panel (**d**) is in °C and applies to both panels (**b**) and (**c**).

**Figure 6:**
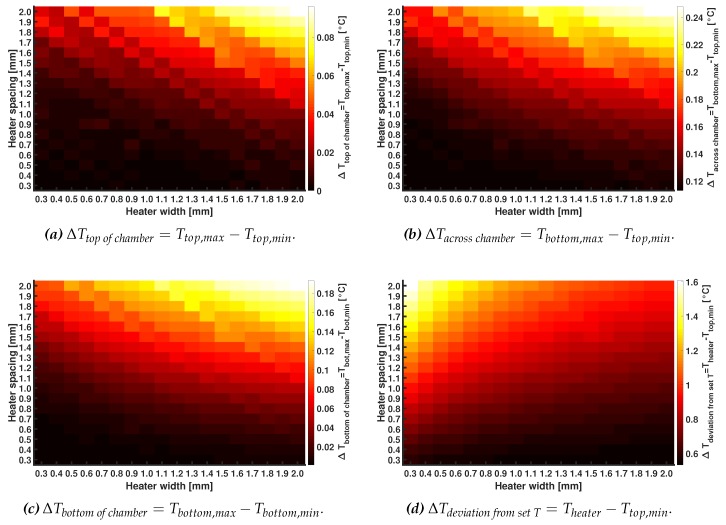
Three temperature differences within the system. In panel (**a**) the $\Delta T_{\mathrm{top}\;\mathrm{of}\;\mathrm{chamber}}$, in panel (**b**) the $\Delta T_{\mathrm{across}\;\mathrm{chamber}}$, in panel (**c**) the $\Delta T_{\mathrm{bottom}\;\mathrm{of}\;\mathrm{chamber}}$, and in panel (**d**) the $\Delta T_{\mathrm{deviation}\;\mathrm{from}\;\mathrm{set}\;T}$ are shown for different heater widths (0.3 mm to 2.0 mm, in the columns) and heater spacings (0.3 mm to 2.0 mm, in the rows). The differences are obtained using a parametric sweeps for both the heater width and heater spacing in the COMSOL Multiphysics 5.3a finite element method heat transfer simulations. The cells in dark indicate the smallest ΔT and the cells in white the largest ΔT.

In Figure 5, the results of heat transfer simulations of two different heater spacings are shown. The heater width for both geometries is 0.3 mm, while the heater spacing in Figure 5b,c are 0.3 mm and 2.0 mm, respectively. In Figure 6a–d, tables with the results of the full parametric sweep for different heater widths and heater spacings are shown. Figure 6a shows the temperature deviation between the highest and lowest temperature at the top of the chamber, i.e., the second H_2_O and COC interface ($\Delta T_{\mathrm{top}\;\mathrm{of}\;\mathrm{chamber}}=T_{top,max}-T_{top,min}$). Figure 6b shows the deviation between the highest and lowest temperature inside the chamber, i.e., between the two COC and H_2_O interfaces ($\Delta T_{\mathrm{across}\;\mathrm{chamber}}=T_{bottom,max}-T_{top,min}$). Figure 6c shows the temperature deviation between the highest and lowest temperature at the bottom of the chamber, i.e., the first COC and H_2_O interface ($\Delta T_{\mathrm{bottom}\;\mathrm{of}\;\mathrm{chamber}}=T_{bot,max}-T_{bot,min}$). Figure 6d shows the deviation between the set heater temperature of 30 °C and the lowest temperature at the top of the chamber, i.e., the second H_2_O and COC interface ($\Delta T_{\mathrm{deviation}\;\mathrm{from}\;\mathrm{set}\;T}=T_{heater}-T_{top,min}$). As can be seen from the results in Figure 6, a combination of small heater widths and heater spacings will result in smaller temperature differences inside the reaction mixture. This is evident as smaller heater spacings will result in a better coverage of the heated area by heater material. The smaller heater widths will result in a smaller heater cross-sectional area, and thus can be operated at lower powers, as is evident from Equation (1). Resulting in the fact that a densely packed meander structure with small heater widths and small heater spacings can dissipate more heat into the system.

Based on these results and its simplicity, a meandering heater pattern of a heater with a width of 0.3 mm and a spacing of 0.3 mm in between the lines is designed. A side note on the chosen heater width and heater spacing is that according to the simulations, the temperature differences within the chamber are less than ±0.3 °C for the most unfavorable dimensions. This temperature difference is still well-accepted in the temperature window in which the MDA reaction takes place (25 °C to 35 °C). However, as pointed out, a smaller cross-sectional area will result in a lower power consumption and therefore these dimensions are chosen. It is known that a meandering heater structure does not give the optimal temperature distribution over the device [26]. Therefore, the heater lines are longer than the width of the reaction chamber, and thus also covering the bulk material outside the chamber in order to improve the temperature uniformity inside the reaction mixture.

**Figure 7:**
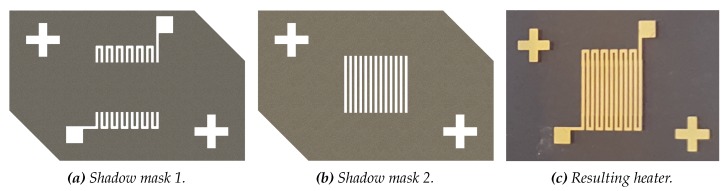
Panels (**a**) and (**b**) show the SolidWorks drawings of shadow masks 1 and 2, respectively. Panel (**c**) shows the resulting metal track after deposition. The crosses are used for alignment under an optical microscope.

The heater pattern is divided over two shadow masks to minimize the length of the narrow Mo tracks in between the meandering structure. This prevents curvature due to intrinsic stresses. See Figure 7 for the outlines of both shadow masks, together with the resulting pattern on COC. The use of two shadow masks will give a metal track in which small parts has the double thickness. Here, the temperature will be lower. The system is designed such that these thicker parts are outside the reaction chamber and temperature control chamber region.

### 3.2. Fabrication

#### Milling and Optical Transparency

**Figure 8:**
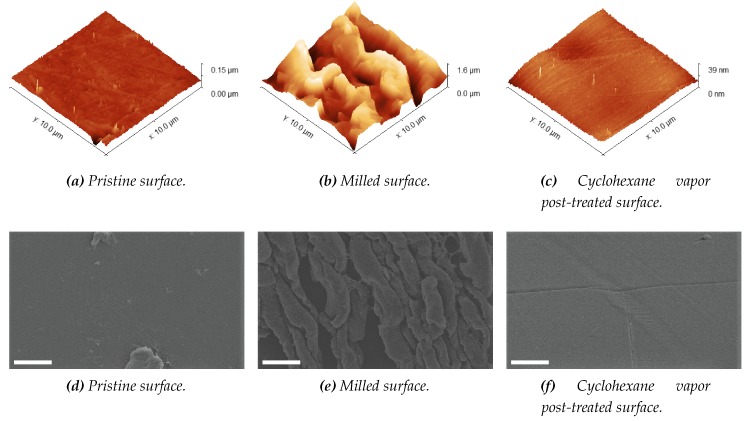
AFM measurements (**a**–**c**) and SEM images (**d**–**f**) showing the effect of a cyclohexane vapor post-treatment on the surface roughness of milled COC. Panel (**a**,**d**) shows the pristine COC surface. (**b**,**e**) Shows the milled surface. (**c**,**f**) Shows the cyclohexane vapor post-treated surface. The scale bars in the SEM images are 2 μm.

The milling increased the surface roughness of the COC plates also increases the surface area. Inhibition of the amplification can be caused by large surface areas as the used chemicals have more surface to have interaction with [44]. The created surface roughness is visualized using a FEI Sirion high resolution scanning electron microscope (HR-SEM) (FEI Company, Hillsboro, OR, USA) and measured using a Bruker Icon Dimension AFM in tapping mode with Bruker Tespa-V2 cantilevers (Bruker Nano Surfaces, Santa Barbara, CA, USA) and Gwyddion 2.52 open source freeware [62]. The results are shown in Figure 8. The surface roughness of pristine COC had a $R_{RMS}$ of 3.5 nm. This increased two orders of magnitude after milling ($R_{RMS}$ of 310.1 nm). With the reported surface treatment [44,59] we were capable of decreasing the surface roughness to a value even lower than that of pristine COC and the lowest reported in literature ($R_{RMS}$ of 0.9 nm). For this grade of COC (TOPAS 6017) it worked the best to do four short exposures of 5 s, with N_2_ blow drying after each exposure, instead of one longer exposure, as is more common in other grades of COC [44,59]. The difference in duration for the cyclohexane vapor post-treatment can be explained by the different ratios of the copolymers present in each grade. As the grade number increase, the ratio changes towards more norbornene monomers and less linear ethene monomers. The norbornene is more apolar due to the bridged cyclic hydrocarbon present in its molecular structure and therefore, will dissolve faster in non-polar solvents, like cyclohexane (vapor).

**Figure 9:**
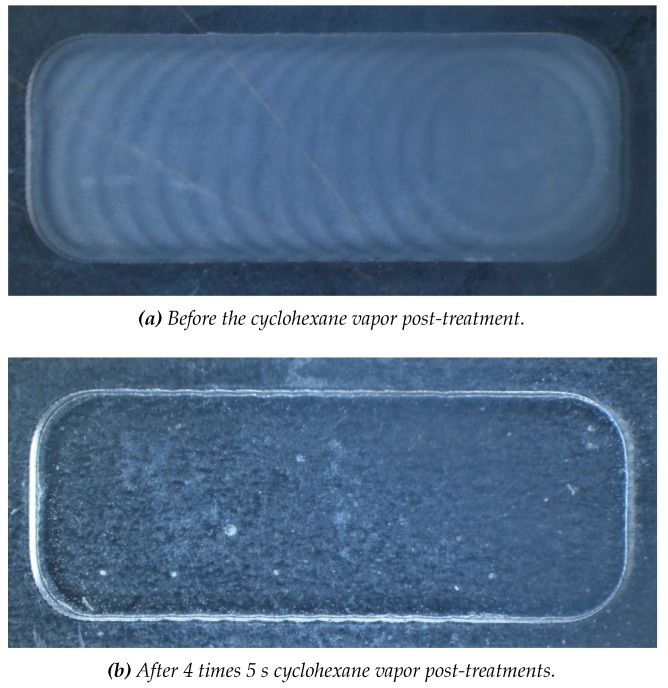
The effect of the cyclohexane vapor post-treatment on the optical transparency of milled COC. (**a**) shows a milled surface and (**b**) the same surface, but post-treated with cyclohexane vapor. The graph in Figure 10 shows the transmittance data of these substrates.

**Figure 10:**
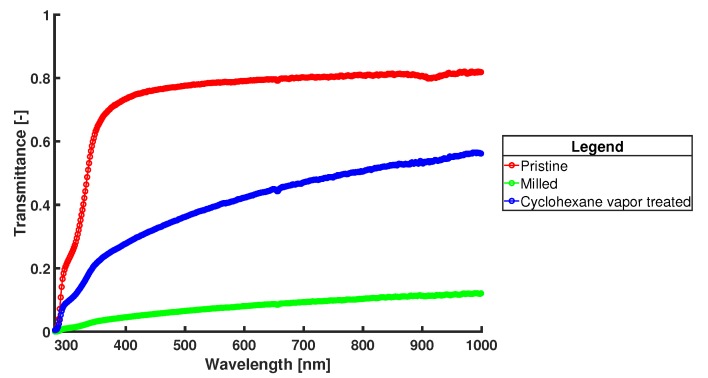
Graph of the transmittance data for a pristine substrate, and the substrates of Figure 9a,b showing the improvement in optical transparancy after cyclohexane vapor post-treatment. The red line is for pristine COC, the green line for milled COC, and the blue line for cyclohexane vapor post-treated COC. Measurements are done with a Woollam M-2000UI ellipsometer in transmittance mode.

Lowering the surface roughness also increased the optical transmittance fivefold. Transmittance measurements in the visible range are done using a Woollam M-2000UI ellipsometer (J.A. Woollam Co., Lincoln, NE, USA). The results can be seen in Figure 9 and Figure 10. Having a high optical transparency in the visible range can be desired when in situ fluorescence detection will be implemented (e.g., EvaGreen fluorescence dye has an excitation wavelength of 500 nm and emission wavelength of 525 nm [63]). However, as in situ fluorescence detection is not used yet in this system and can also be done through the transparent PCR plate sealing foil, no further effort is put into optimizing this procedure to get even better optical transmittance.

### 3.3. Metal Adhesion

To get reliable heaters, four possible options are investigated for their adhesion properties to the COC substrate. The adhesion of Au and Pt deposited by either evaporation or DC magnetron sputtering is investigated using the Scotch tape test [60,61] before and after temperature cycling up to 100 °C. Test patterns consisting of rectangular metal strips of 2 mm by 14 mm are fabricated by depositing 100 nm of metal using a hand-made shadow mask made out of DuPont Kapton^®^ HN polyimide film of 0.05 mm thickness (RS Components B.V., Haarlem, The Netherlands). See Table 4 for the results of the Scotch tape test.

Normally, heating up a glass or Si substrate with thin metal strips while measuring the resistance ($R_{T}$) in these metal strips at certain temperature intervals (*T*) yields directly a linear relation, which can be fitted with $R_{T}\;/\;R_{0}=1+\alpha \left(T-T_{0}\right)$ [64], in which α is the the temperature coefficient of resistance (TCR) value. The thin-film TCR values have to be measured as they differ from the bulk TCR values due to its dependency on layer purity, grain size, and deposition method [65,66]. Belser and Hicklin also lists other attributes, such as surface roughness, porosity, and adsorbed materials present in or on the substrate which could influence the TCR value [64]. The bulk TCR values are 0.0034 K^−1^ and 0.0037 K^−1^ for Au and Pt [67].

**Figure A2:**
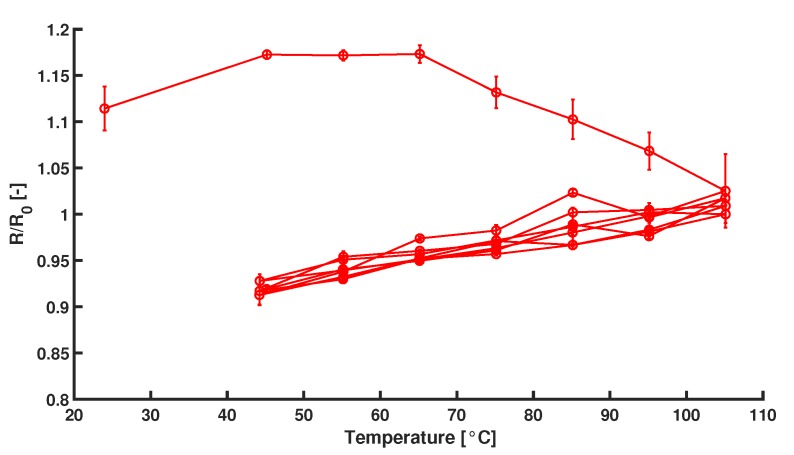
Thermal annealing of an evaporated 100 nm Au layer.

The TCR characterizations of the metal strips on a COC substrate did not yield trustworthy TCR values at the first cycle. The first temperature cycle can be seen as a kind of thermal annealing, and therefore gives an hysteresis in the graphs, as can be seen in Figure A2 in Appendix C.1. After this first cycle, the values more or less show the linear behavior. The resulting TCR of this linear part is in agreement with the TCR ranges of Belser and Hicklin [64] and is given in Table 4. Belser and Hicklin used for their experiments substrates with coefficients of linear thermal expansion lower than 1.2 × 10^−5^ °C^−1^ [64]. The coefficient of linear thermal expansion for Au and Pt are 1.42 × 10^−5^ K^−1^ and 0.88 × 10^−5^ K^−1^, respectively [68]. COC of the grade TOPAS 6017 has a coefficient of linear thermal expansion of 6.0 × 10^−5^ K^−1^ [42]. This mismatch in coefficients of linear thermal expansion can give strain in the metal layers. Both Au [69,70,71] and Pt [72,73,74] are used as strain-sensitive gauges, and thus are sensitive to strain-induced geometry changes due to thermal expansion.

**Figure A3:**
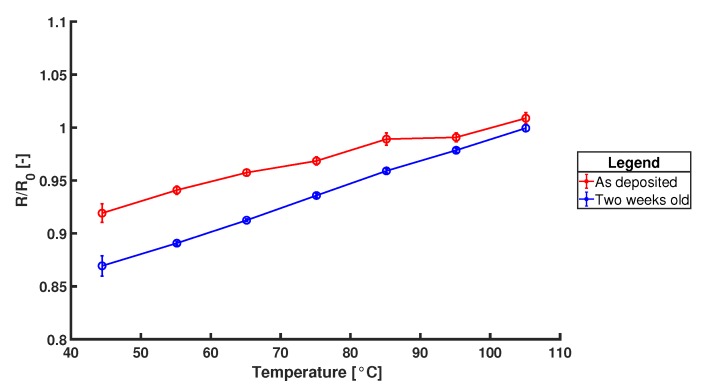
The effect of two weeks aging in ambient conditions on a 100 nm Au layer. The TCR of the as deposited layer is 0.00161 K^−1^ and the TCR of the two week old layer is 0.00224 K^−1^.

Another effect influencing the TCR value of the metal layer is aging. As can be seen in Figure A3 in Appendix C.2, the TCR value already changes after two weeks storing in ambient conditions. This could be due to adsorbed materials present on the surface [64].

However, in this device, the TCR is not of importance as the metal structure will not be used as temperature sensor. Real-time temperature sensing is done using a thermocouple in the temperature monitor chamber. The resistance of the heater structure changes with temperature; thus, the dissipated power changes when a fixed voltage or current is used. However, the results in Section 3.4 show a 25 h stability test with a constant input potential and only a ±1.5 °C deviation. The TCR can become more important when other (higher) temperatures are required for the amplification.

Based on the results in Table 4, the choice of heater material and deposition method to be used in the actual device is Au deposited using sputtering. Sputtering is an industrial-scale technique that is already being used in, for example, the car mirror and headlight industry [75].

### 3.4. Chip Functioning

**Figure 11:**
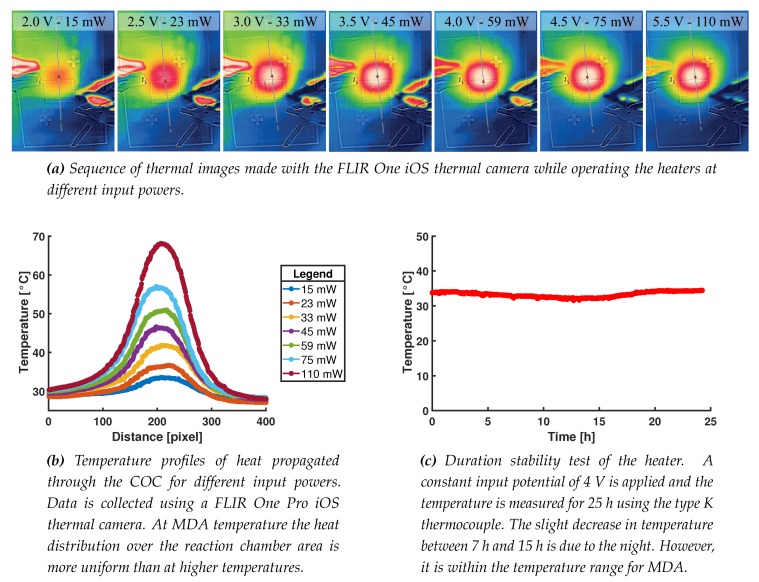
Characterization of the 100 nm thick Au heater structure deposited by sputtering using the two sequential shadow masks. Panel (**a**) depicts the thermal images on the other side of the 1.5 mm thick substrate, while operating the heater at different input powers. (**b**) The graph with the recorded heat profiles of panel (**a**). Panel (**c**) shows the durability test in which a constant potential is applied for 25 h.

Characterization of the actual heat distribution is done using a FLIR One Pro iOS thermal camera (FLIR Systems, Inc., Wilsonville, OR, USA). Thermal images of the heat distribution are made at the side of the substrate without the resistor, whereas different input powers are used to heat up the heater. Au reflects the infrared radiation of the environment directly, therefore an image with the resistor facing the camera would give a heat map of the surrounding and not of the real temperature of the heater. These measurements also gives a better insight of the heat distribution inside the reaction chamber. The images are processed using the FLIR postprocessing freeware. Results of these measurements are shown in Figure 11a,b. The results are in good agreement with the estimations in Table 1. The slight deviation between the values can be explained by the fact that the heated area in the calculations had an assumed value, the thermal camera measurements used 1.5 mm thick COC substrates without a water-filled chamber, the actual resistors have small parts wich have a double thickness due to the two used shadow masks, and rounding of the values used in the calculations.

The reliability of the heater is tested by inserting the thermocouple into the temperature control chamber (see Figure 3a). A constant input potential of 4 V is applied using the Keithley source and the temperature is measured for 25 h. This exceeds the required operation time at least twelve-fold, meaning that it is a good indication for the reliability of the heater and thermocouple. The results are shown in Figure 11c.

**Figure 12:**
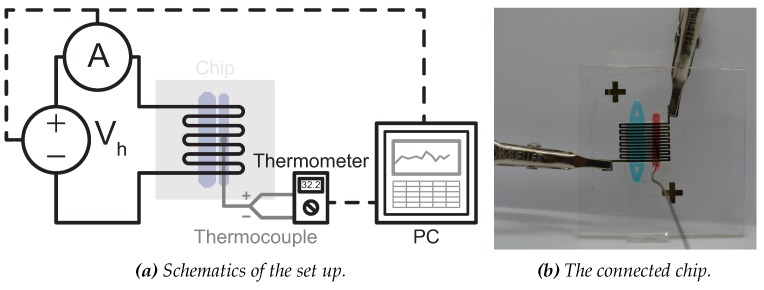
The set up for MDA reacions, (**a**) the schematics in which the voltage source is a Keithley SourceMeter, the thermocouple is connected to a Tenma thermometer. (**b**) The DNA amplification chip (both the amplification chamber and temperature control chamber are filled with food coloring dye for visualization purposes).

To perform on-chip amplifications, the resistive heater on the chip is connected to the Keithley source using crocodile connections and the thermocouple is inserted in the temperature control chamber and connected to a Tenma 72-7715 Thermometer (see Figure 12).

### 3.5. DNA Amplification

**Figure 13:**
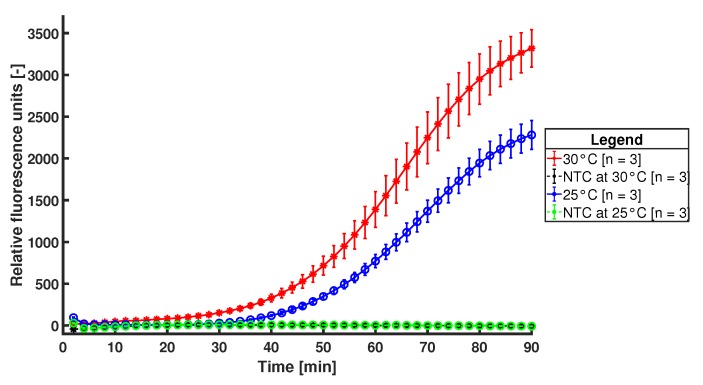
Fluorescence signal of MDA reactions performed at 25 °C and 30 °C, together with their (overlapping) NTCs.

First, to determine the temperature window of operation, MDA reactions are performed at 25 °C and 30 °C using the Illustra GenomiPhi V2 DNA amplification kit and EvaGreen fluorescence dye. From the literature, we know that this reaction does not work above 35 °C due to degradation of the protein activity in presence of a substrate [44]. In Figure 13, a graph of the fluorescence signal during MDA reactions at 25 °C and 30 °C, together with their non template control (NTC) is shown. These reactions are carried out in a conventional Bio-Rad CFX96 Touch Real-Time PCR machine (Bio-Rad Laboratories, Inc., Hercules, CA, USA) and the results show that the chosen proof-of-principle DNA amplification reaction is temperature dependent to some extent, but that there is a wide range of temperatures at which the amplification can be performed, i.e., 25°C to 35°C. This makes the functioning of the integrated resistive heater less critical than the stability shown in Figure 11c.

MDA reactions are also performed inside an Eppendorf tube (Eppendorf AG, Hamburg, Germany) and inside the chip, again using the Illustra GenomiPhi V2 DNA amplification kit and EvaGreen fluorescence dye. As heat supply the water bath of an IKA Rotary Evaporator RV 8V (IKA-Werke, Staufen im Breisgau, Germany) is used. This water bath is according to its specification stable within a range of the set temperature ±0.1 °C. The chip and an Eppendorf tube are loaded with the reaction mixture containing the DNA sample and the EvaGreen dye solution. Here, the Eppendorf tube is serving as a control to show that the fabrication steps of the chips are not inhibiting the MDA reaction. The inlet and outlet of the chip are sealed with the Microseal “B” PCR plate sealing foil. The closed chip and tube are heated up in a separate water bath to 95 °C and kept at that temperature for 3 min to denaturate the dsDNA. Subsequently, the chip and tube are cooled down by placing it in an ice bath for 5 min after which the rest of the reagents are added. The complete mixtures are according to Table A3 in Appendix D. After closing the chip and tube again, they are placed in the water bath of the rotary evaporater and left there for 90 min, after which the reaction is terminated at 65 °C.

**Figure 14:**
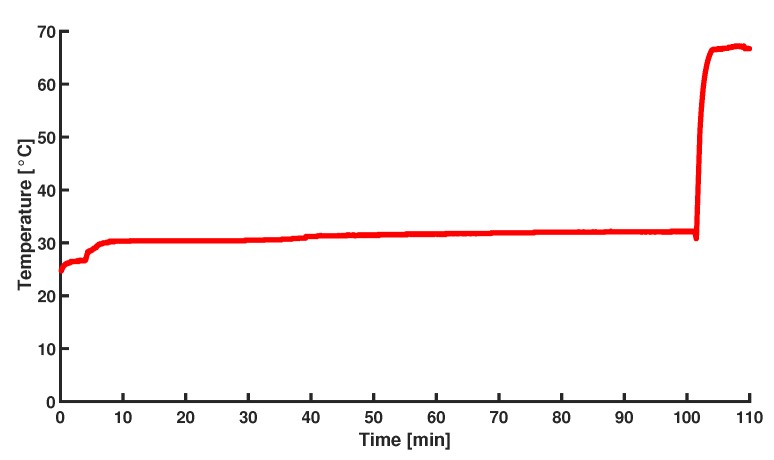
Temperature profile recorded with the thermocouple inside the water-filled temperature control chamber during the on-chip MDA amplification.

The MDA is also performed inside the chip, but with the integrated Au resistive heater serving as heat source. The set up shown schematically in Figure 12. The same procedure is followed as with the water bath heated test. Denaturation is done in a separate water bath. The heater is driven by an input potential of 3.2 V to get to a temperature of 30 °C and at the end of the reaction, the system is heated up to 65 °C by applying a potential of 9.2 V in order to terminate the amplification. In Figure 14 the logged temperature during the amplification is shown.

**Figure 15:**
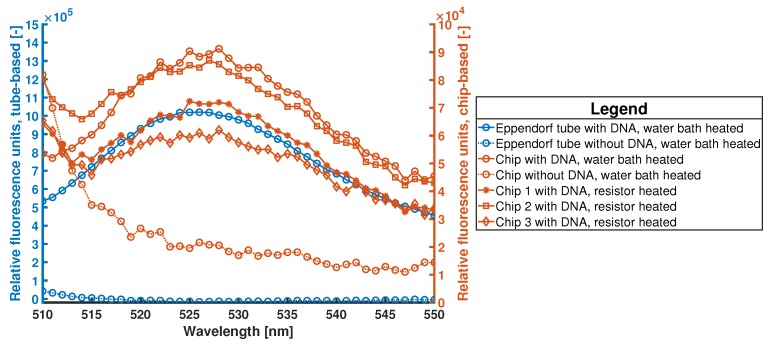
Fluorescence signal of DNA-binded EvaGreen dye after MDA reactions. The reactions are performed inside an Eppendorf tube and on-chip. As heating, two different methods are used, namely a water bath (for both the Eppendorf tube and chip) and the on-chip resistive heater (3 chips). The fluorescence intensity cannot be used as a value to quantify the amount of DNA [44]. The two blue lines with circular points are the MDA (continuous line) and NTC (dashed line) performed inside an Eppendorf tube. The two orange lines with circular points are the MDA (continuous line) and NTC (dashed line) performed inside a water bath heated chip. The three continuous orange lines with asterisks, squares, and diamonds represent the signals obtained after MDA reactions performed inside chips while using the integrated heater.

**Figure A4:**
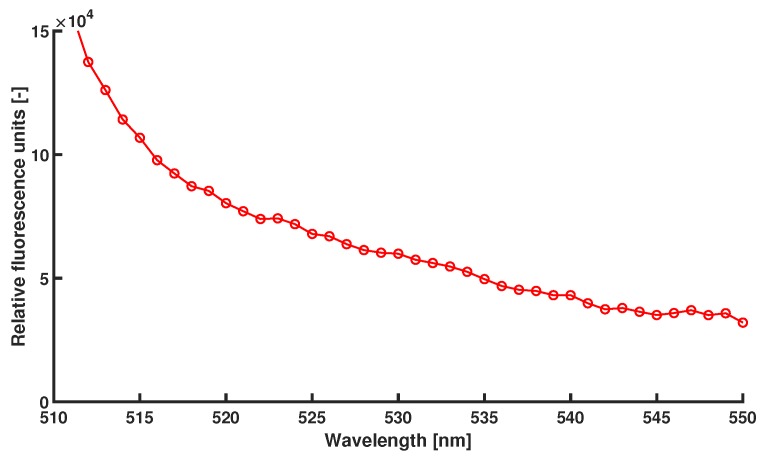
The background signal of the mixture in Table A4.

After the amplifications, the reaction mixtures are pipetted out of the chips and tubes and into 1 mL quartz cuvettes containing 55 μL MilliQ DI water (Merck Millipore, Burlington, MA, USA). Fluorescence measurements are done in a Horiba Scientific FluoroMax+ spectrofluorometer to verify each amplification. The mixture is excitated at a wavelength of 500 nm and the emission spectrum is measured at wavelengths from 510 nm to 550 nm (bounded EvaGreen dye has a peak at 525 nm [63]). The measured spectra are normalized by subtracting the background signal of a mixture containing only the reaction buffer, the sample buffer, EvaGreen, and DNA. No Enzyme was added to this mixture, therefore no amplification could take place. See Figure 15 for the results obtained in the Eppendorf tube and chips. Figure A4 in Appendix E shows the background signal which is subtracted from all measurements.

As can be seen in Figure 15, the spectra of the amplification performed inside the chip, and by applying heat with the water bath as well as with the integrated Au-resistive heater, show the same trend as the amplification performed in the Eppendorf and heated by water bath. There is an order of magnitude difference in the fluorescence signal. However, the fluorescence intensity cannot be used as a value to quantify the amount of DNA. EvaGreen is a bis-intercalating cyanine fluorescence dye consisting of two monomeric DNA-binding dyes which are linked by a flexible spacer. These two DNA-binding dyes bind each in between two base pairs, which make them simple and fast, but also nonuniform and non-specific [44,63]. However, with this dye, a simple yes-or-no answer can be obtained if the amplification took place, as can be seen in Figure 15.

## 4. Conclusions

The aim of this study was to fabricate biocompatible, low-cost, and disposable chips with integrated heater, which should be able to perform DNA amplification, and possible in situ fluorescence detection in the near future. In this case there is no interest in quantification of the DNA, but only in amplification of DNA until the detection threshold is reached. As proof-of-principle the MDA reaction and ex-situ fluorescence measurements were used.

With the proposed fabrication process, low-cost and biocompatible chips (Figure 12b) were fabricated. The integrated resistive heaters on the chips were characterized and showed a temperature stability of ±2 °C over a time period of 25 h, which is at least twelve-fold longer than the required operating times for DNA amplification reactions [6,8,9,10,11]. The main cause of this period of lowered temperature was due to the fact that the measurement was run overnight.

With the proof-of-principle device, successful DNA amplifications using MDA inside a disposable polymeric chip were achieved. The heat for the reaction was applied using the integrated low-cost Au-resistive heater. The device was operated at a suitable temperature for MDA reactions and the amplified DNA was measured using EvaGreen fluorescence dye and an ex situ spectrofluorometer. A distinct peak is visible in the reaction mixtures which is absent in the NTC mixtures. The operating temperature for MDA reactions is around 30 °C, which is comparable with a nice summer day. Using amplification reactions which such low reaction temperatures could encounter problems at warmer locations. However, as MDA is not sequence specific, this reaction will not be integrated in the final protocols. MDA was only used as proof-of-principle reaction to show the biocompatibility of the device and functioning of the integrated heater. Sequence specific amplifications, e.g., HDA and LAMP, are performed at higher temperatures, as will be discussed in Section 5. This makes the system less sensitive to the hot summer days.

The device in its current state is not fully conform the WHO-SDI ASSURED criteria [49] as it still relies on the use of (expensive) external equipment. However, the first steps are made to an ASSURED device. Future steps which will make the device fully ASSURED are given in the next section (Section 5).

## 5. Outlook

Future steps, which will result in a device for early-stage detection of, for example, zoonoses, include studies on the optimization of this device for sequence specific DNA amplifications (e.g., primer design and reaction optimization), i.e., HDA or LAMP. HDA utilizes DNA helicase (an enzyme also used in vitro during DNA replication) to separate the dsDNA instead of thermal denaturation. After separation, ssDNA binding proteins hybridize on the ssDNA strands for stabilization, ensuring that the next primer will have time to bind to the ssDNA stripe and a DNA polymerase will extend the primers with the complementary bases. This method is a truly isothermal technique in which the separation of the dsDNA can be performed at the same temperature as the amplification reaction, i.e., 64 °C [9]. LAMP is more similar to MDA in the way it also uses heat to denature the dsDNA. After denaturation, a set of four primers (six can be used as well to achieve better selectivity) and a DNA polymerase is used at isothermal conditions (65 °C) to amplify the DNA [11]. When used in combination with reverse transcriptase, LAMP becomes a RNA amplification method, which could be used for RNA-containing viruses [11], like virus-based zoonoses diseases as the corona viruses [22]. Despite not being a truly isothermal technique, LAMP offers the possibility to use turbidity as detection method [76]. Such a detection method would simplify the required equipment even further as a decrease in transmitted light through the chip can be used as detection method.

Different amplification techniques require different temperatures. Based on Table 1 one can conclude that a higher temperature would also give a larger temperature gradient within the system. This can be disadvantageous for amplification reactions, as optimal denaturation temperatures are in the range 92 °C to 94 °C [77]. The denaturation in this research was done in a separate water bath, so this temperature gradient was circumvented. However, when on-chip denaturation and/or another amplification technique will be used, a second step will be the optimization of the heater in order to create better temperature uniformity within the system. This can be done by using different heater geometries [26,37] or using double-sided heating [28].

The third step that has to be optimized in the sample collection and work-up procedure. One has to think of what kind of samples to collect in order to have the biggest chance of having the agent of the disease present in that sample (i.e., blood, mucus, saliva, etc.). Such crude samples contain full cells, with the DNA present within. There are different approaches to perform cell lysis in order to extract the DNA [78]. Various components of bodily fluids, and reagents and products of the lysis are well-known to inhibit the amplification reaction [79]. However, MDA [80] and HDA [9] could also be performed on crude samples.

A fourth step in the near-future is the development of a first prototype with all hardware integrated in a single piece of equipment. Such a device in its pure essence will consist of a battery to power the heater and detection, a chip holder to firmly keep the chip in its place, a thermocouple for real-time monitoring of the temperature, and a LED light and a photodiode. The lamp and photodiode could both be used for fluorescence measurements and turbidity measurements.

## Figures and Tables

**Table 1:** Calculated required heater temperatures and powers when a desired temperature at the top of the chamber is assumed. The calculations are based on Equations (2) and (3).

Desired Temperature	Heater Temperature	Required Power
[°C]	[°C]	[mW]
30	31.7	17.3
54	59.1	58.6
65	71.8	77.7
72	79.8	89.6
95	106.3	129.4

**Table 2:** Specifications of simulation computer.

Component	Hardware
Motherboard	ASUS RoG Rampage VI Extreme
CPU	Intel Core i9 7900X processor, 10 cores, 3.3 GHz
CPU cooler	NZXT Kraken X62 AM4 water cooling
RAM	G.Skill Trident Z RGB LED 4 × 16 GB DDR4, 3200 MHz
GPU	Radeon Pro WX2100, 2 GB
Power supply	Corsair HX1000i 1000 W

**Table 3:** Estimated current densities for the 4 extreme heater geometries.

$s_{\mathrm{heater}}$	$w_{\mathrm{heater}}$	$l_{\mathrm{heater}}$	$I_{\mathrm{Au}}$	$I_{\mathrm{Au}}\;/\;A_{\mathrm{cross}-\mathrm{section}}$	$I_{\mathrm{Pt}}$	$I_{\mathrm{Pt}}\;/\;A_{\mathrm{cross}-\mathrm{section}}$
[mm]	[mm]	[mm]	[mA]	[10^9^ A m^−2^]	[mA]	[10^8^ A m^−2^]
0.3	0.3	134.9	32	1.06	14	4.73
0.3	2.0	46.4	54	1.84	24	8.06
2.0	0.3	30.9	172	8.60	77	3.83
2.0	2.0	22.2	203	1.01	90	4.51

**Table 4:** Results of the Scotch tape metal adhesion tests before and after heat cycling. Here, + means passing the tape test, − means failing the tape test, and +/− means that not all test structures failed the tape test.

Material	Method	Tape Test		Initial	TCR
		Before	After	Resistance [Ω]	K^−1^
Au	Evaporation	+	−	5.0	0.00161
Au	Sputtering	+	+	8.2	0.00192
Pt	Evaporation	+	+	400 ${}^{*}$	−0.00440 ${}^{*}$
Pt	Sputtering	+	+/−	6.8	0.00207

* These results are not reliable as the shadow mask gave contamination in the metal track.

## Abbreviations

The following abbreviations are used in this manuscript:

AFM	atomic force microscopy
Au	gold
CAD	computer-aided design
CAM	computer-aided manufacturing
CNC	computer numerical control
COC	cyclic olefin copolymer
DC	direct current
DI	de-ionized
DNA	deoxyribonucleic acid
dsDNA	double stranded DNA
H_2_O	water
HDA	helicase-dependent amplification
LAMP	loop-mediated isothermal amplification
MDA	multiple displacement amplification
Mo	molybdenum
N_2_	nitrogen
NTC	non template control
PCR	polymerase chain reaction
PDMS	polydimethylsiloxane
PID	proportional-integral-derivative
Pt	platinum
PVD	physical vapor deposition
RNA	ribonucleic acid
SEM	scanning electron microscopy
ssDNA	single stranded DNA
SDI	Sexually Transmitted Diseases Diagnostics Initiative
TCR	temperature coefficient of resistance
$T_{g}$	glass transition temperature
WGA	whole genome amplification
WHO	World Health Organization

## Appendix B. Equations and Values Used in the COMSOL Multiphysics Study

### Appendix B.1. Water

For H_2_O, the build-in temperature-dependent equations for the density ($\rho_{H_{2}O}$, see equations in Equation (A1)), heat capacity at constant pressure ($C_{P,H_{2}O}$, see equations in Equation (A2)), and thermal conductivity ($\kappa_{H_{2}O}$, see equations in Equation (A3)) are used. The ratio ($\gamma_{H_{2}O}$) of the specific heats at constant pressure ($C_{P,H_{2}O}$) and constant volume ($C_{V,H_{2}O}$) is calculated manually according to the table from the Engineering Toolbox website [81] and Equation (A4). The values for $\gamma_{H_{2}O}$ are listed in Table A1.

(A1) $$\begin{array}{c} \rho_{H_{2}O}\left(T\right)=\left\{\begin{array}{cc} 972.7584+0.2084T-4\times 10^{-4}T^{2} & \;\mathrm{for}\;273\leq T<283\\ 345.28+5.749816T-0.0157244T^{2}+1.264375\times 10^{-5}T^{3} & \;\mathrm{for}\;283\leq T<373 \end{array}\right. \end{array}$$

(A2) $$\begin{array}{cc} C_{P,H_{2}O}\left(T\right)= & 12010.1471-80.4072879T+0.309866854T^{2}\;\;\mathrm{for}\;273.15\leq T<553.75\\ \; & -5.38186884\times 10^{-4}T^{3}+3.62536437\times 10^{-7}T^{4} \end{array}$$

(A3) $$\begin{array}{cc} \kappa_{H_{2}O}\left(T\right)= & -0.869083936+0.00894880345T-1.58366345\times 10^{-5}T^{2}\;\mathrm{for}\;273.15\leq T<553.75\\ \; & +7.97543259\times 10^{-9}T^{3} \end{array}$$

(A4) $$\gamma_{H_{2}O}=\frac{C_{P,H_{2}O}}{C_{V,H_{2}O}}$$

**Table A1:** The ratio of specific heats of water.

*T*	$\gamma_{H_{2}O}$	*T*	$\gamma_{H_{2}O}$
[K]	[-]	[K]	[-]
273.16	1.000592782	393.15	1.157465496
283.15	1.001073729	413.15	1.199809492
293.15	1.006591292	433.15	1.246234334
298.15	1.010560913	453.15	1.297534537
303.15	1.015203400	473.15	1.355013713
313.15	1.025996023	493.15	1.420794975
323.15	1.038520763	513.15	1.498241758
333.15	1.052405261	533.15	1.592792563
343.15	1.067512483	553.15	1.714447794
353.15	1.083658241	573.15	1.883524402
363.15	1.100748613	593.15	2.148448797
373.15	1.118756966	613.15	2.666580033
383.15	1.137648990	633.15	4.550527720

### Appendix B.2. Cyclic Olefin Copolymer

Values for the density ($\rho_{COC}$ = 1020 kg m^−3^), specific heat ($c_{COC}$, see Table A2), and thermal conductivity ($\kappa_{COC,\;23\;{}^{{}^{\circ}}C}$ = 0.17 W m^−1^ K^−1^ and $\kappa_{COC,\;320\;{}^{{}^{\circ}}C}$ = 0.24 W m^−1^ K^−1^, linear fit in between these point) of TOPAS 6017 COC are obtained via TOPAS Advanced Polymers (TOPAS Advanced Polymers, Farmington Hills, MI, USA). The heat capacity at constant pressure ($C_{P,COC}$) is calculated assuming a homogeneous body of mass *m*, via Equation (A5), where COMSOL interpolated linearly in between the points.

(A5) $$C_{P,COC}=c_{COC}\times m$$

**Table A2:** The specific heat of TOPAS 6017 COC.

*T*	*c*	*T*	*c*
[°C]	[W m^−1^ K^−1^]	[°C]	[W m^−1^ K^−1^]
30	1333	180	2298
70	1538	210	2412
110	1754	250	2539
150	1968	290	2645
160	2047	330	2758

Convective heat loss to the air is also taken into account with Equation (A6), which is often used in simulations [56].

(A6) $$h=10\left[W\;m^{-2}K^{-1}\right]$$

## Appendix C. TCR Measurements

The two subsections below show two effects on the TCR measurements, i.e., the effect of thermal annealing during the first temperature cycle and the effect of aging.

### Appendix C.1. Thermal Annealing

The graph below is an example of the measured thermal annealing of a metal layer on COC. Here, only the graph for evaporated Au is shown, but all except evaporated Pt show this behavior. Evaporated Pt had some contaminants in the metal track. This is most probably caused by the shadow mask.

### Appendix C.2. Aging

The layer analyzed in Figure A2 is stored for two weeks in ambient conditions and the TCR is analyzed again. From the graph it is visible that the aging has some effect on the TCR of the metal layer.

## Appendix D. MDA Reaction Mixture

The reaction mixture for the on-chip MDA reaction consisted of the following.

**Table A3:** On-chip MDA reaction mixtures.

Sample	Reaction Buffer	Sample Buffer	DNA	MilliQ DI Water	EG	Enzyme
	[μL]	[μL]	[μL]	[μL]	[μL]	[μL]
Tube with DNA	9	9	1	-	4	1
Tube NTC	9	9	-	1	4	1
Chip with DNA	9	9	1	-	4	1
Chip NTC	9	9	-	1	4	1

## Appendix E. Background Signal Fluorescence Measurements

All fluorescence measurements in the Horiba Scientific FluoroMax+ spectrofluorometer are normalized by subtracting the background signal. This background signal is measured using the mixture in Table A4.

**Table A4:** Mixture used for background signal measurement.

Sample	Reaction Buffer	Sample Buffer	DNA	MilliQ DI Water	EG	Enzyme
	[μL]	[μL]	[μL]	[μL]	[μL]	[μL]
Background	9	9	1	1	4	-
